# How to define and quantify a bad death in palliative home care? Across-sectional and exploratory study using Canadian interRAI data

**DOI:** 10.1186/s12904-025-01720-7

**Published:** 2025-03-20

**Authors:** Amanda Mofina, Nicole Williams, John P. Hirdes, Gary Cheung, James Downar, Kieran L. Quinn, Dawn M. Guthrie

**Affiliations:** 1 School of Public Health Sciences, University of Waterloo, Waterloo, ON Canada; 2 Department of Kinesiology and Physical Education, Wilfrid Laurier University, Waterloo, ON Canada; 3 Department of Psychological Medicine, School of Medicine, The University of Auckland, Auckland, New Zealand; 4 Division of Palliative Care, Department of Medicine, University of Ottawa and Bruyere Research Institute, Ottawa, ON Canada; 5 Temmy Latner Centre for Palliative Care, Department of Medicine, Sinai Health System, University of Toronto, Toronto, ON Canada

**Keywords:** Bad death, Pain, Depression, Loneliness, InterRAI

## Abstract

**Background:**

Dying is a complex process comprised of physical, social, cultural, spiritual, environmental, and interpersonal relationship factors that contribute to both good and bad death experiences. Bad deaths have historically been explored with a qualitative lens. This study aimed to identify key indicators of a bad death and examine predictors for each indicator using population-level data.

**Methods:**

This cross-sectional study analyzed routinely collected clinical and sociodemographic data using the Resident Assessment Instrument for Home Care (RAI-HC) between April 2007 and March 2020. 16,586 home care clients aged 18 years and older who died and had an assessment completed within 30 days of their death were included. Four indicators of a bad death were examined: self-reported loneliness, severe depressive symptoms, daily pain that is horrible or excruciating, and pain that is severe/excruciating and uncontrolled by medications. These indicators were interRAI specific variables that captured common bad death constructs in the existing literature. The study sample was separated into groups based on these four indicators and each individual could populate more than one group. Chi-square analyses were used to examine the relationship between potential risk factors and each bad death indicator.

**Results:**

Of the total sample, 50.9% were 85 + years of age, and 54.7% were female. The prevalence of experiencing at least one of the bad death indicators was 33.5%. Each indicator significantly increased the likelihood of experiencing one of the other indicators with the ORs ranging from 1.70 to 3.26. Other important predictors that increased the odds of experiencing each bad death indicator included: any psychiatric diagnoses (OR range: 1.29–1.89), experiencing conflict with family or friends (OR range: 1.21–3.40), and a decline in social interaction which was distressing to the person (OR range: 2.06–3.70).

**Conclusions:**

These four bad death indicators were common among community-dwelling adults. This study found that there was an interconnectedness between the bad death indicators. Clinically, the relationship between these indicators means that addressing one aspect of a bad death may positively influence the others. Early identification of these issues, along with client and family collaboration, can aid in optimizing the likelihood of a good death.

## Background

Death and dying are unique experiences for every person [1–3]. It is well established that advancements in medicine have coincided with improved end-of-life care and the emergence of the concepts of a good and bad death [1]. A good death experience is often comprised of a variety of social, cultural, spiritual, environmental, and interpersonal relationship factors [1, 2, 4]. A good death is therefore difficult to universally define and operationalize, as it will likely look different for everyone or every culture. Similarly, defining the factors that contribute to a bad death are highly variable.

A bad death is generally understood as a negative health event or deleterious health experience that occurs prior to end-of-life. In a scoping review Wilson and Hewitt [1] identified most factors contributing to a bad death experience could be categorized into experiences of pain and suffering, sudden/unexpected deaths, prolonged deaths, disrespect of the dying person, and dying without dignity. More broadly, characteristics of a bad death can span across areas of physical health (e.g., pain, physical decline and loss of function, and prolonged death), psychological health (e.g., depression, anxiety), spiritual and religious health (e.g., dignity, wishes not being carried out, existential loneliness/suffering), and social health (e.g., isolation, dying alone) [1, 3, 5–14]. Consideration of these factors, and their relative importance to someone, are integral in shaping and facilitating a client-centred palliative care plan that is highly individualized. A good death is therefore not simply the absence of a bad death; however, there are some common factors identified in the literature that span across multiple health domains that contribute to a bad death. The factors that are commonly used in defining what constitutes a bad death were derived from a body of literature that focused heavily on qualitative methods wherein care providers’ and patients’ perspectives were considered. In the forementioned scoping review, 80% of the studies were qualitative, and only one of 25 included articles was quantitative [1]. These studies were conducted across various health care settings including the community, hospice, hospitals, and nursing homes, with the majority being conducted in the United States.

The factors that have been identified as possible indicators of a bad death also align with some of the palliative care quality indicators (QIs) that have been developed for identifying and assessing quality of care in community-based palliative care services using existing interRAI data. interRAI is the name of an international group of researchers and clinicians that aim to improve care for persons with disabilities and medical complexities through the development and implementation of a suite of health instruments. These health instruments (e.g., The Resident Assessment Instrument for Home Care (RAI-HC)) are comprised of a set of items that capture the broad determinants of health, as well as generate key outputs such as outcome measures, care planning protocols, and quality indicators [15–18]. Specifically, there are QIs that capture pain (prevalence, worsening, and improvement), loneliness and mood-related aspects of health, aspects of social engagement, and caregiver distress [19]. These QIs are outcome measures used at the population-level, often for continuous quality improvement initiatives. They look back in time at the rates for a group of individuals and are therefore not used to guide individual-level care planning. While it is important that these factors are considered in quality improvement initiatives, there is a gap in proactively identifying individuals at risk for experiencing a bad death.

There is existing Canadian evidence on symptom trajectories among those with life-limiting illnesses during the final six months of life that are congruent with risk factors for a bad death experience [12, 14, 20]. The symptoms that are aligned with bad death indicators include: pain, functional decline and dependency on others, and caregiver distress. This is an important connection because it provides insight into a method of identifying and operationalizing individual-level bad death indicators within a broader population.

There are commonalities between the factors identified in constituting a bad death, health symptoms at the end-of-life, and QI outcome measures. There is an evidence gap with respect to predictors of common factors associated with a bad death. A better understanding of these predictors could better support care planning and decision-making at the client-level. Therefore, the key objectives of the current study were to: (1) explore the prevalence across a set of potential indicators of a bad death at the population level in Canada; and (2) examine the predictors associated with each of these potential indicators as being important components of a bad death using data collected with the Resident Assessment Instrument for Home Care (RAI-HC). The RAI-HC is a mandated home care health assessment that captures information on physical and cognitive functioning, psychosocial supports, and social health. The RAI-HC is part of a suite of assessments used along the continuum of care that aim to provide clinical information, evidence-informed care planning, information regarding resource utilization, and care quality to facilitate health care service that is client-centred [17]. This work represents the initial stages in the development and validation of an electronic algorithm to flag individuals who may be at an increased risk of a bad death. This type of clinical algorithm would support home care clinicians in their decision-making as they develop individualized care plans and determine who could potentially benefit from early palliative care interventions.

## Methods

### Data source

This cross-sectional study analyzed secondary data collected using the RAI-HC between April 2007 and March 2020. The RAI-HC was fully completed across Ontario, Newfoundland and Labrador, and Yukon Territory, with partial use of the instrument in British Columbia (all of British Columbia except Northern Health) and Alberta (all of Alberta except Calgary Zone) [5]. The assessment is mandated or recommended for use in Canada by each province/territory and is completed for clients expected to receive services for 60 days or longer. The RAI-HC is completed by a trained assessor (typically a registered nurse), by assessing multiple sources of information, including input from the client, informal caregivers, and available health records, to guide their clinical judgement in completing the assessment. The study sample included home care clients aged 18 years and older who died and had a RAI-HC assessment completed within 30 days of their death (*n* = 16,586). If an individual had more than one assessment 30 days before death, the most proximal assessment prior to death was used. Existing literature has highlighted that some observed adverse outcomes tend to increase slightly during the final four weeks preceding death (e.g., pain, health instability, depression), which is why a 30-day timeframe was chosen [12–14]. Overall, missing data accounted for less than 6% across all analyses. There was 5.1% of missing data across the Caregiver Risk Evaluation (CaRE) algorithm because some individuals do not have a caregiver and the algorithm is not generated when there are missing data. Additionally, there was 3.7% missing data related to Instrumental Activities of Daily Living (IADL) performance, which is an item used in the IADL scale, and similar to the algorithms, a scale score cannot be generated when an item is missing. Missing data was therefore not a concern, given the low rates and justification for the missing data. This study was reviewed and approved by the Research Ethics Board at Wilfrid Laurier University (REB #6977) and the need for individual-level consent was waived.

### Indicators of a bad death

We examined four potential indicators of a bad death, namely, self-reported loneliness, severe depressive symptoms, pain that is horrible or excruciating, and pain that is severe/excruciating and medications do not control it. These four factors were used as potential indicators of a bad death as they are often highlighted within the existing literature [1, 3, 5–14], and are congruent with existing population-level quality indicators among seriously ill or palliative clients [19]. The study sample was separated into groups, based on these four indicators. Each individual could populate one or more of these indicator groups, since they were not mutually exclusive. It was important to allow individuals to populate multiple indicator groups because these indicators are rarely experienced in isolation among those with life limiting illnesses.

Self-reported loneliness was captured on the RAI-HC as a dichotomous variable (yes/no). Having severe depressive symptoms was determined using the Depression Rating Scale (DRS) [21], a validated 14-point additive scale that is generated using seven mood and behaviour items. A cut-point of five or higher has been shown to indicate moderate to severe depressive symptoms [21–24]. The RAI-HC has several items measuring pain over the previous three days. To create the group of persons experiencing horrible and excruciating daily pain, a composite dichotomous (yes/no) variable was created. For example, those who experienced both daily pain AND experienced pain intensity that was severe or horrible or excruciating were included in this group (yes). Similarly, another composite dichotomous (yes/no) variable was created for the second pain indicator. In this case, those who had both severe/excruciating pain AND their medications were not adequately controlling their pain were included in this group (yes).

### Independent predictors of a bad death

Other health index scales embedded within the RAI-HC, which are automatically generated upon completion of the assessment, were also considered among factors related to the dying experience. These scales capture multiple broader health determinants and across all scales, a higher value indicates a greater level of impairment.

1. The Activity of Daily Living Self-Performance Hierarchy (ADL-SHS) scale categorizes ADL decline and functional loss in stages; early loss (e.g., hygiene), middle loss (e.g., toileting and locomotion), and late loss (e.g., eating). The scores range from zero (independent) to six (total dependence) [25]. A cut-point of two or greater was used to indicate at least mild functional impairment.
2. The Instrumental Activities of Daily Living (IADL) Involvement Scale is a measure of functional performance across seven IADLs (e.g., meal preparation, housework) and scores range from 0 to 21 [25]. A score of 14 + was used to indicate a greater level of impaired functioning when performing these tasks [26]. Both the ADL-SHS and the IADL involvement scale are valid and reliable measures of functional ability [27].
3. The Cognitive Performance Scale (CPS) score is derived from items reflecting the person’s functional capabilities in the areas of short-term memory, cognitive skills for daily decision-making, expressive communication, and independence in eating. The CPS score ranges from zero (no impairment) to six (severe impairment), where a cut-point of two or higher was used to indicate at least mild impairment in cognitive functioning [28]. The CPS has excellent inter-rated reliability, has been validated against the Mini Mental State Examination, [29] and is correlated with the Montreal Cognitive Assessment [30].
4. The Caregiver Risk Evaluation (CaRE) algorithm is a decision support tool that was developed to identify caregivers at risk of experiencing caregiver burden [31]. It categorizes informal caregivers into one of four independent groups: low, moderate, high, and very high risk of experiencing caregiver burden. The algorithm has some evidence of predictive validity as home care clients whose caregivers are in the very high risk group are significantly more likely to be admitted to a long-term care home [31].

Additionally, demographic characteristics (e.g., age, sex, marital status), disease diagnoses (e.g., dementia other than Alzheimer’s dementia, Alzheimer’s dementia, multiple sclerosis, Parkinson’s disease, arthritis, any psychiatric diagnoses), and multimorbidity which was defined by a chronic disease count dichotomized at the median (0–4 chronic conditions versus 5 chronic conditions or more), social measures (e.g., changes in social activity, conflict with family or friends, loneliness), measures of pain, and prognosis were also considered in this study as potential predictors of a bad death.

### Analysis

Each of the four bad death indicators were analyzed independently using chi-square tests to examine relationships across each categorical variable considered as a potential predictor. Due to the large sample size, there was clear evidence of type I error. As such, we shifted our focus to odds ratios (ORs), and the associated 95% confidence intervals, when determining statistical and clinical significance. An odds ratio showing a 20% change (i.e., OR of 1.20 or higher or an OR of 0.83 or less) was used to indicate statistical significance and clinical importance of the variable [32]. Variables that were considered important predictors for each bad death indicator were then summarized and unadjusted ORs were presented. It is important to note that chi-square analyses were not used for comparisons across the bad death indicators as these groups were not mutually exclusive. As such, comparisons across each of these four groups are dependent on proportions alone. Additionally, it is also important to highlight the use of each of these statistical approaches because they influence the interpretation, which is exploratory in nature, and aimed to elucidate the complexity and interconnectedness of both predictors and bad death indicators. To examine the complex relationship between each of the bad death indicators, the analyses included these indicators as potential predictors of the other indicators.

All analyses were performed using SAS software (version 9.4) [33]. The study followed the STrengthening the Reporting of OBservational studies in Epidemiology (STROBE) guidelines [34].

## Results

Of the total sample, 50.9% were 85 + years of age, 54.7% were female, 39.2% were married, and 33.5% experienced at least one indicator of a bad death. The age variable was not normally distributed, therefore median and interquartile range were reported for each indicator. The prevalence across the four potential indicators of a bad death ranged from 8.8% (*n* = 1,465) for horrible or excruciating pain that was not controlled by medications, 12.2% (*n* = 2,029) for self-reported loneliness, 13.3% (*n* = 2,203) for severe depressive symptoms, and 17.5% (*n* = 2,904) for experiencing horrible or excruciating pain (Table 1).

**Table 1:** Overall sample demographic characteristics (*N* = 16,586)

Variable	% (*n*)
**Age**	
**Median (IQR)**	83.0 (13.0)
18–64	8.1 (1339)
65–74	11.8 (1953)
75–84	29.2 (4849)
85+	50.9 (8445)
**Sex**	
Female	54.7 (9067)
Male	45.3 (7518)
**Marital Status**	
Never Married	4.4 (737)
Married	39.2 (6506)
Widowed	41.3 (6855)
Separated/Divorced	6.5 (1075)
Other	8.5 (1413)
**Education**	
Less than high school	33.5 (5555)
High school	16.4 (2711)
Some college or university/technical/ trade school	13.4 (2223)
Post-secondary	9.7 (1605)
Unknown	27.1 (4492)
**Disease Diagnosis (present)**	
Alzheimer’s dementia	7.0 (1158)
Other type of dementia	24.3 (4033)
Multiple sclerosis	0.7 (115)
Parkinson’s disease	4.9 (806)
Cancer	26.5 (4391)
Congestive heart failure	26.5 (4387)
Chronic obstructive pulmonary disease	27.6 (4571)
Renal failure	15.3 (2533)
Stroke	20.4 (3378)
Coronary artery disease	33.0 (5480)
Arthritis	51.7 (8573)
Hemiplegia	2.5 (408)
Any psychiatric diagnosis^a^	16.0 (2648)
**Multimorbidity**	
0–4	49.8 (8264)
5+	50.2 (8321)
**Activities of Daily Living Self-Performance Hierarchy Scale**	
Independent/minor supervision (0–1)	27.3 (4523)
Moderate/severe dependence (2+)	72.7 (12063)
**Instrumental Activities of Daily Living (IADL) Involvement Scale**	
None/minor difficulty (0–13)	20.6 (3284)
Moderate/major difficulty (14+)	79.5 (12697)
**Cognitive Performance Scale (CPS)**	
No/mild cognitive challenges (0–1)	30.2 (5014)
Moderate/severe cognitive challenges (2+)	69.8 (11572)
**Caregiver Risk Evaluation (CaRE)**	
Low	16.4 (2580)
Moderate	22.7 (3579)
High	39.2 (6165)
Very high	21.7 (3419)
**Indicators of a Bad Death**	
**Severe signs and symptoms of depression**	
No (DRS score between 0–4)	86.7 (14383)
Yes (DRS score of 5 or higher)	13.3 (2203)
**Self-reported Loneliness**	
No	87.8 (14557)
Yes	12.2 (2029)
**Daily Pain that is severe**,** excruciating**,** or horrible**	
No	82.5 (13682)
Yes	17.5 (2904)
**Pain that is severe/excruciating and not managed with medications**	
No	91.2 (15121)
Yes	8.8 (1465)

^a^ This includes signs/symptoms of depression as well as any other type of psychiatric diagnosis

The four groups were very similar across basic demographic and clinical characteristics, which was expected given that these groups were not mutually exclusive. Medical comorbidities were common in individuals who experienced a bad death, as measured by any of the four indicators: 33.0% had coronary artery disease, 51.7% arthritis, 26.5% had cancer, 27.6% had chronic obstructive pulmonary disease (COPD) and 26.5% had congestive heart failure (Table 1). The high prevalence of different medical diagnoses in this cohort demonstrates that end-of-life care services are provided to individuals with a various health conditions that extend beyond cancer. High or very high risk of caregiver burden was observed in 64.5–88.6% of individuals who experienced a bad death, as measured by the CaRE algorithm across the four indicators (Table 2).

**Table 2:** Demographic, diagnostic, and clinical characteristics of individuals across the four indicators of a bad death

Variable	self-reported loneliness		Experienced severe depressive symptoms		Experienced pain that was horrible or excruciating		Experienced uncontrolled pain	
Column % (*n*)	No (*n* = 14,557)	Yes (*n* = 2,029)	No (*n* = 14,383)	Yes (*n* = 2,203)	No (*n* = 13,682)	Yes (*n* = 2,904)	No (*n* = 15,121)	Yes (*n* = 1465)
**Median age (IQR)**	83.0 (13.0)	83.0 (13.0)	83.0 (13.0)	81.0 (14.0)	84.0 (12.0)	81.0 (16.0)	83.0 (13.0)	81.0 (17.0)
**Age (years)**								
18–64	8.0 (1160)	8.8 (179)	7.5 (1083)	11.6 (256)	6.9 (938)	13.8 (401)	7.5 (1131)	14.2 (208)
65–74	11.8 (1711)	11.9 (242)	11.4 (1632)	14.6 (321)	11.2 (1530)	14.6 (423)	11.5 (1732)	15.1 (221)
75–84	29.5 (4300)	27.1 (549)	28.9 (4155)	31.5 (694)	29.3 (4009)	28.9 (840)	29.2 (4421)	29.2 (428)
85+	50.7 (7386)	52.2 (1059)	52.2 (7513)	42.3 (932)	52.7 (7205)	42.7 (1240)	51.8 (7837)	41.5 (608)
**Sex**								
Male	46.2 (6730)	38.8 (788)	46.1 (6627)	40.4 (891)	46.1 (6307)	41.7 (1211)	45.7 (6905)	41.9 (613)
Female	53.8 (7826)	61.2 (1241)	53.9 (7755)	59.6 (1312)	53.9 (7375)	58.3 (1692)	54.3 (8216)	58.1 (851)
**Marital status**								
Never married	4.3 (632)	5.2 (105)	4.5 (643)	4.3 (94)	4.4 (599)	4.8 (138)	4.4 (665)	4.9 (72)
Married	42.1 (6127)	18.7 (379)	39.0 (5615)	40.4 (891)	39.1 (5354)	39.7 (1152)	39.1 (5915)	40.3 (591)
Widowed	39.7 (5776)	53.2 (1079)	41.8 (6015)	38.1 (840)	41.8 (5715)	39.3 (1140)	41.6 (6286)	38.8 (569)
Separated/ divorced	6.0 (877)	9.8 (198)	6.2 (887)	8.5 (188)	5.9 (813)	9.0 (262)	6.3 (949)	8.6 (126)
Other	7.9 (1145)	13.2 (268)	8.5 (1223)	8.6 (190)	8.8 (1201)	7.3 (212)	8.6 (1306)	7.3 (107)
**Education**								
Less than high school	32.7 (4766)	38.9 (789)	33.5 (4819)	33.4 (736)	33.5 (4583)	33.5 (972)	33.6 (5083)	32.2 (472)
High school	16.1 (2337)	18.4 (374)	16.3 (2338)	16.9 (373)	16.2 (2222)	16.8 (489)	16.3 (2457)	17.3 (254)
Some college or university/technical/ trade school	13.4 (1943)	13.8 (280)	13.5 (1940)	12.9 (283)	13.4 (1827)	13.6 (396)	13.4 (2024)	13.6 (199)
Post-secondary	9.9 (1438)	8.2 (167)	9.8 (1403)	9.2 (202)	9.8 (1337)	9.2 (268)	9.7 (1468)	9.4 (137)
Unknown	28.0 (4073)	20.7 (419)	27.0 (3883)	27.6 (609)	27.1 (3713)	26.8 (779)	27.0 (4089)	27.5 (403)
**Disease diagnosis (present)**								
Alzheimer’s dementia	7.5 (1091)	3.3 (67)	7.3 (1056)	4.6 (102)	7.8 (1060)	3.4 (98)	7.3 (1110)	3.3 (48)
Other type of dementia	24.8 (3608)	21.0 (425)	24.2 (3485)	24.9 (548)	26.2 (3578)	15.7 (455)	25.2 (3814)	15.0 (219)
Multiple sclerosis	0.7 (96)	0.9 (19)	0.7 (101)	0.6 (14)	0.7 (90)	0.9 (25)	0.7 (103)	0.8 (12)
Parkinson’s disease	5.0 (728)	3.8 (78)	4.9 (702)	4.7 (104)	5.1 (699)	3.7(107)	5.0 (761)	3.1 (45)
Cancer	27.2 (3961)	21.2 (430)	26.4 (3794)	27.1 (597)	25.3 (3460)	32.1 (931)	25.9 (3912)	32.7 (479)
Congestive heart failure	25.9 (3770)	30.4 (617)	26.4 (3791)	27.1 (596)	26.5 (3620)	26.4 (767)	26.4 (3996)	26.7 (391)
Chronic obstructive pulmonary disease	26.5 (3850)	35.5 (721)	27.1 (3903)	30.3 (668)	27.3 (3734)	28.8 (837)	27.4 (4138)	29.6 (433)
Renal failure	15.1 (2192)	16.8 (341)	15.1 (2167)	16.6 (366)	14.8 (2021)	17.6 (512)	15.1 (2284)	17.0 (249)
Stroke	20.4 (2974)	19.9 (404)	20.6 (2957)	19.1 (421)	21.0 (2878)	17.2 (500)	20.8 (3139)	16.3 (239)
Coronary artery disease	32.6 (4745)	36.2 (735)	33.1 (4756)	32.9 (724)	32.7 (4480)	34.5 (1000)	32.8 (4960)	35.5 (520)
Arthritis	50.3 (7316)	62.0 (1257)	51.2 (7362)	55.0 (1211)	49.8 (6819)	60.4 (1754)	50.7 (7660)	62.4 (913)
Hemiplegia	2.5 (356)	2.6 (52)	2.5 (353)	2.5 (55)	2.4 (331)	2.7 (77)	2.4 (366)	2.9 (42)
Any psychiatric diagnosis^a^	14.8 (2148)	24.6 (500)	n/a	n/a	15.3 (2091)	19.2 (557)	15.7 (2366)	19.3 (282)
**Multi-morbidity**								
0–4	51.0 (7416)	41.8 (848)	50.6 (7282)	44.6 (982)	50.6 (6929)	46.0 (1335)	50.2 (7584)	46.5 (680)
5+	49.1 (7140)	58.2 (1181)	49.4 (7101)	55.4 (1220)	49.4 (6753)	54.0 (1568)	49.8 (7537)	53.5 (784)
**Depression Rating Scale (DRS)**								
No/moderate signs/symptoms of depression (0–4)	88.9 (12941)	71.1 (1442)	n/a^b^	n/a^b^	88.9 (12166)	76.3 (2217)	88.0 (13300)	73.9 (1083)
Severe signs or symptoms of depression (5+)	11.1 (1616)	28.9 (587)	n/a^b^	n/a^b^	11.1 (1516)	23.7 (687)	12.0 (1821)	26.1 (382)
**Activities of Daily Living Self-Performance Hierarchy Scale (ADL-SHS)**								
Independent/minor supervision (0–1)	26.3 (3833)	34.0 (690)	28.3 (4067)	20.7 (456)	27.6 (3774)	25.8 (749)	27.3 (4133)	26.6 (390)
Moderate/severe dependence (2+)	73.7 (10724)	66.0 (1339)	71.7 (10316)	79.3 (1747)	72.4 (9908)	74.2 (2155)	72.7 (10988)	73.4 (1075)
**Instrumental Activities of Daily Living (IADL) Involvement Scale**								
None/minor difficulty (0–13)	19.4 (2733)	28.9 (551)	21.3 (2947)	15.8 (337)	20.0 (2632)	23.1 (652)	20.2 (2940)	24.0 (344)
Moderate/major difficulty (14+)	80.6 (11340)	71.1 (1357)	78.7 (10906)	84.2 (1791)	80.0 (10530)	76.9 (2167)	79.8 (11610)	76.0 (1087)
**Cognitive Performance Scale (CPS)**								
No/mild cognitive challenges (0–1)	30.1 (4379)	31.3 (635)	31.3 (4500)	23.3 (514)	28.9 (3959)	36.3 (1055)	29.7 (4487)	36.0 (527)
Moderate/severe cognitive challenges (2+)	69.9 (10178)	68.7 (1394)	68.7 (9883)	76.7 (1689)	71.1 (9723)	63.7 (1849)	70.3 (10634)	64.0 (938)
**Caregiver Risk Evaluation (CaRE)**								
Low	15.1 (2103)	25.8 (477)	17.2 (2341)	11.4 (239)	16.6 (2152)	15.5 (428)	16.4 (2349)	16.4 (231)
Moderate	24.5 (3400)	9.7 (179)	26.2 (3579)	n/a^c^	24.5 (3176)	14.6 (403)	23.7 (3401)	12.7 (178)
High	37.8 (5245)	49.8 (920)	36.5 (4978)	56.7 (1187)	37.4 (4855)	47.3 (1310)	38.1 (5467)	49.6 (698)
Very high	22.7 (3147)	14.7 (272)	20.2 (2752)	31.9 (667)	21.5 (2791)	22.7 (628)	21.8 (3120)	21.3 (299)

^a^ This includes signs/symptoms of depression as well as any other type of psychiatric diagnosis. ^b^ It is not appropriate to fill these cells in as they are measuring the same construct in the same way, with the same cut-off scores. ^c^ Individuals with a DRS of 5 + cannot populate the moderate group (moderate group can only have a DRS of 2 or lower)

A main finding when examining these four indicators of a bad death, was that each indicator was associated with higher odds of experiencing each of the other indicators. The ORs ranged from 1.70 to 3.26, and all of them would be considered clinically relevant based on our cut-point. For example, those who experienced severe symptoms of depression had 3.26 higher odds of experiencing loneliness (OR = 3.26; CI: 2.92, 3.64), 2.49 higher odds of experiencing daily pain that was horrible or excruciating (OR = 2.49; CI: 2.25, 2.75), and 2.58 higher odds of experiencing uncontrolled pain (OR = 2.58; CI: 2.27, 2.92) compared with those who did not experience severe symptoms of depression (Table 3).

**Table 3:** variables with a clinically relevant^a^ relationship to the four indicators of a bad death

Variable	Experienced self-reported loneliness (*n* = 2,029)	Experienced severe depressive symptoms (*n* = 2,203)	Experienced pain that was horrible or excruciating (*n* = 2,904)	Experienced uncontrolled pain (*n* = 1,465)
	**Odds ratio (95% confidence interval)** ^b^			
**Age (years)**				
18–64	Reference			
65–74	0.92 (0.75, 1.13)	0.83 (0.69, 1.00)	0.65 (0.55, 0.76)	0.69 (0.57, 0.85)
75–84	0.83 (0.69, 0.99)	0.71 (0.60, 0.83)	0.49 (0.43, 0.56)	0.53 (0.44, 0.63)
85+	0.93 (0.78, 1.10)	0.53 (0.45, 0.61)	0.40 (0.35, 0.46)	0.42 (0.36, 0.50)
**Sex**				
Male	Reference			
Female	1.35 (1.23, 1.49)	1.26 (1.15, 1.38)	1.20 (1.10, 1.30)	1.17 (1.05, 1.30)
**Disease diagnosis (reference = absence of the diagnosis)**				
Alzheimer’s dementia	0.42 (0.33, 0.54)	0.61 (0.50, 0.76)	0.42 (0.34, 0.51)	0.43 (0.32, 0.57)
Other type of dementia	0.80 (0.72, 0.90)	1.04 (0.93, 1.15)	0.53 (0.47, 0.58)	0.52 (0.45, 0.61)
Multiple sclerosis	1.43 (0.87, 2.34)	0.91 (0.52, 1.59)	1.31 (0.84, 2.05)	1.21 (0.66, 2.20)
Parkinson’s disease	0.76 (0.60, 0.96)	0.97 (0.78, 1.19)	0.71 (0.58, 0.88)	0.60 (0.44, 0.81)
Arthritis	1.61 (1.47, 1.77)	1.17 (1.07, 1.28)	1.54 (1.42, 1.67)	1.61 (1.45, 1.80)
Any psychiatric diagnosis	1.89 (1.69, 2.11)	n/a	1.32 (1.19, 1.46)	1.29 (1.12, 1.48)
**Multi-morbidity**				
0–4	Reference			
5+	1.45 (1.32, 1.59)	1.27 (1.16, 1.39)	1.21 (1.11, 1.31)	1.16 (1.04, 1.29)
**Conflict with family or friends**				
No	Reference			
Yes	1.72 (1.53, 1.93)	3.40 (3.06, 3.77)	1.21 (1.08, 1.35)	1.23 (1.07, 1.42)
**Change in social activities**				
No decline	Reference			
Decline, not distressed	0.80 (0.72, 0.90)	1.30 (1.16, 1.45)	1.11 (1.01, 1.21)	1.10 (0.97, 1.25)
Decline and distressed	2.41 (2.15, 2.71)	3.70 (3.29, 4.16)	2.06 (1.85, 2.29)	2.13 (1.86, 2.45)
**Caregiver Risk Evaluation (CaRE)** ^c^				
Low	Reference			
Moderate	0.23 (0.19, 0.28)	n/a	0.64 (0.55, 0.74)	0.53 (0.44, 0.65)
High	0.77 (0.69, 0.87)	2.34 (2.02, 2.71)	1.36 (1.20, 1.53)	1.30 (1.11, 1.52)
Very high	0.38 (0.33, 0.45)	2.37 (2.03, 2.78)	1.13 (0.99, 1.30)	0.98 (0.81, 1.17)
**Prognosis of less than 6 months to live**				
No	Reference			
Yes	0.54 (0.47, 0.63)	1.22 (1.09, 1.36)	1.53 (1.39, 1.69)	1.44 (1.27, 1.64)
**Self-reported loneliness**				
No	Reference			
Yes	n/a	3.26 (2.92, 3.64)	1.70 (1.52, 1.90)	1.73 (1.51, 2.00)
**Severe symptoms of depression**				
No	Reference			
Yes	3.26 (2.92, 3.64)	n/a	2.49 (2.25, 2.75)	2.58 (2.27, 2.92)
**Pain is horrible or excruciating**				
No	Reference			
Yes	1.70 (1.52, 1.90)	2.49 (2.25, 2.75)	n/a	n/a
**Medications adequately control pain**				
No pain/pain is controlled	Reference			
Pain is not controlled	1.73 (1.51, 2.00)	2.58 (2.27, 2.92)	n/a^d^	n/a

^a^ Significant findings were defined as an odds ratio that was either > = 1.2 or < = 0.83. ^b^ All ORs were calculated with the absence of the indicator as the reference group. ^c^ Individuals scoring 5 + on the DRS cannot populate the moderate risk category of the CaRE algorithm. ^d^ The OR could not be calculated since one of the cells had a count of less than 10

In terms of demographic characteristics, both age and sex seemed to play an important role. Increased age had a protective effect across all four bad death indicators compared to those aged 18–64. Being female significantly increased the odds of experiencing loneliness (OR = 1.35; CI: 1.23, 1.49), depressive symptoms (OR = 1.26; CI: 1.15, 1.38), and daily excruciating pain (OR = 1.20; CI: 1.10, 1.30) compared to males. The group experiencing severe depressive symptoms experienced the highest ORs across all other indicators of a bad death (self-reported loneliness (OR = 3.26), pain is horrible or excruciating (OR = 2.49), and pain is not well controlled by medications (OR = 2.58), as well as across items capturing caregivers at high or very high risk of experiencing caregiver burden (OR = 2.34 and 2.37 respectively), decline in social interaction which was distressing (OR = 3.70), and conflict with family or friends (OR = 3.40). Similarly, individuals with any psychiatric diagnoses had increased odds of experiencing indicators of a bad death compared to those without a psychiatric diagnosis. Those with any psychiatric diagnoses had 1.89 increased odds of experiencing loneliness, 1.32 increased odds of experiencing pain that was horrible or excruciating, and 1.29 increased odds of experiencing pain that is not well controlled by medication. Conversely, neurological conditions (i.e., Alzheimer’s dementia, other types of dementia, and Parkinson’s disease) had a predominantly protective effect across the bad death indicators (Table 3).

## Discussion

To our knowledge, this is the first Canadian study to look at potential indicators of a bad death among home care clients who were nearing end-of-life. In this large cross-sectional study, roughly one-third of clients experienced one or more of the four potential indicators of a bad death, highlighting the significance of flagging these issues. In keeping with previous literature, older age is negatively correlated with both pain and depression [13, 14, 35–38]. Irrespective of age, we also found that the presence of each indicator was associated with a higher likelihood of each of the other indicators, further evidence of the complexity of the dying experience, and the potential drawback to addressing them independently. Given the interconnection of these clinical issues, addressing one will likely have an important and positive influence on the others.

The four potential indicators of a bad death encompass two important constructs commonly discussed in the palliative care literature, namely, pain and depression [1, 3, 5, 13]. Overall, the prevalence rate of each indicator, in the current study, is comparable to ranges reported in the literature among seriously ill or palliative home care clients (5 − 30%) [13, 23, 39].

The prevalence rate of moderate to severe depressive symptoms was 13.3% in our study, which is very similar to the 10% reported in Canada [40], but slightly lower than that reported among decedents assessed with the interRAI Home Care instrument rate which ranged from 20 to 24% [12–14]. A higher cut-point on the DRS was used when defining depressive symptoms (score of 5+) in the current study compared to previous literature, because the intent was to identify and flag those individuals experiencing more severe symptoms as an indicator of a bad death [12–14, 23]. Depressive symptoms are often underdiagnosed [41], however, they are particularly important to identify in individuals with a life-limiting illness as these symptoms can persist for up to a year among roughly two-thirds of seriously ill home care clients [32]. It is critical to recognize that depressive symptoms are not merely a result of aging or approaching end-of-life [23]. Early identification and intervention can address these symptoms to improve the overall dying experience [23, 40, 42].

The rate of self-reported loneliness, at 12.2%, was consistent with other reports using interRAI Home Care data among clients in Canada assessed within six months of dying [13, 39]. Existing evidence highlights that prevalence rates among older adults, aged 85 and above, increases to nearly 45% [39]. A similar relationship between increased age and self-reported loneliness was observed in our study where among those experiencing loneliness, 52% were aged 85 years and above. This may suggest that self-reported loneliness becomes more prevalent in older adulthood among those with a serious illness as individuals may not be able to interact or socialize with their family and friends in the same capacity. Other areas that were related to loneliness included a diagnosis of arthritis, multimorbidity, and severe signs and symptoms of depression which is consistent with existing literature that examined incident loneliness in home care [39].

Pain is a common symptom experienced by individuals receiving palliative care [43, 44]. Pain affects aspects of daily living including physical functioning, social functioning, and psychosocial and mental well-being [43, 45]. Pain is perhaps the most important indicator of a bad death, as most individuals fear dying in pain [1, 44]. In the current study, roughly 18% of clients experienced horrible or excruciating pain, consistent with the work by Seow and colleagues [14]. They reported a prevalence of between 15% and 20% during the final six months of life among individuals with cancer. The rate increased with proximity to death. It is vitally important to address pain as it can negatively affect not only an individual’s physical functioning, but also their psychosocial well-being and overall quality of life. This further highlights the importance of continuing to screen for and address pain in its entirety for all individuals with a serious or life-limiting illness.

Caregivers were either among the high- or very high-risk groups for experiencing burden among the following bad death indicators: experiencing severe depressive symptoms, experiencing pain that was horrible or excruciating, and experiencing uncontrolled pain. Informal caregivers provide significant support to their loved ones and their care needs (intensity and amount) typically increase with proximity to death [46, 47]. This additional strain may increase the likelihood of caregivers experiencing burden. The evidence supports an inter-relationship between the home care client experiencing depressive symptoms and the caregiver experiencing burden [32, 48], and the current study had similar findings. It is within reason to consider that aspects of caregiver burden (e.g., anger, distress, depression) may affect the mood of the person for whom they are providing care, especially given the complexities of the informal caregiver and client relationship [48]. Among those experiencing self-reported loneliness, there was a protective relationship between caregiver burden and self-reported loneliness, which may be related to level of formal support, level of informal support, and social connections beyond the primary caregiver. Aspects of informal support that may also play a role in caregiver burden include length of time caregiver supports have been in place and consistency among caregiver supports, level of experience as a primary caregiver, and the client’s relationship to the primary caregiver. These types of variables were not available in the current data; however, it would be important to consider in future studies.

Caregiver supports, and level of caregiver experience may also play a role in the trend observed across the two pain indicators wherein there was a 23-32% reduction in the risk of experiencing the pain indicators among the ‘very high’ risk of caregiver burden group compared to the ‘high’ risk group. It is important to note that the odds of experiencing either of the pain indicators in the very high caregiver burden group is not statistically significant. High-quality palliative care is holistic in nature, encompassing both the individual and their family. It is therefore important to identify caregivers at risk of burden, and address their needs as best as possible, to optimize the death experience for the individual receiving home care.

Each of the bad death indicators are potentially modifiable, and many of the risk factors for these indicators may also be amenable to change. Prognosis has typically been used as the benchmark for when to initiate palliative care services and has focused on individuals with a shorter (e.g., < 6 months) prognosis. While we do not know whether the individuals in the current study were receiving palliative care, it is evident that earlier identification and intervention to address the potential indicators of a bad death and/or other modifiable risk factors are paramount in improving end-of-life care and outcomes. Through earlier identification of symptoms, additional care needs may emerge and as such, comprehensive client- and family-centred care can be delivered in a way that aligns with their needs, preferences, and goals. Ideal care planning is dynamic and early discussions aid in better preparation to ensure clients’ wishes are respected throughout their journey to improve the likelihood that they die with dignity. If we can identify these factors associated with a bad death earlier in the illness trajectory, then they can be addressed, thereby increasing the likelihood of a positive experience.

We anticipate that the findings of this study will be useful in creating further applications to support client care, such as a potential algorithm to flag individuals at high risk of a bad death. Specifically, the operationalization of an adverse outcome (i.e., a bad death) and the development of a set of predictors of that outcome (e.g., pain and depressive symptoms), can inform multiple interRAI functions such as the development of risk profiles, care planning, and assessment of quality. The bad death indicators identified in this study could be used in conjunction with existing clinical assessment protocols on pain and mood to create risk profiles of those at higher risk for experiencing a bad death [49]. These risk profiles could play a key role in service planning and provision, and in preventing adverse outcomes.

## Limitations

The cross-sectional nature of the design does not account for the changes in a person’s illness trajectory over time. Since the dataset did not include the clinical notes, only the interRAI assessment items, we could not ascertain whether clients had received a palliative approach to their care. Additionally, there is limited data on service provision and intervention(s) within the interRAI assessment. Although all of this is tracked carefully within the electronic clinal notes, it was unavailable to us. There is also potential for selection bias in this study. Data from individuals who decline home care services, or those who require inpatient acute care due to complex medical needs are not available, which could result in an under-reporting of these issues. Lastly, this study only looked at four key indicators of a bad death and the factors associated with these four bad death indicators. It is important however, to acknowledge that this was a preliminary and exploratory study, to assess and identify key factors contributing to a bad death experience using a comprehensive assessment that is completed routinely within the existing health care system. The findings from this study are foundational in better understanding the factors associated with a bad death and are essential in identifying key interventions for this population.

## Conclusions

This cross-sectional study highlights four possible indicators of a bad death derived from a large dataset of home care clients who died within 30 days of their last assessment including issues like depressive symptoms and pain. Understanding the complex nature of a potential bad death, and the association between the established indicators of a bad death discussed in this study, are both important in helping optimize the likelihood of a good death. This can be achieved by collaborating with the client, and their family, regarding their goals of care, and ideally, intervening when these indicators are identified early in the disease trajectory. The interconnected nature of the outcomes is an advantage, in that clinically addressing one aspect of a bad death has a high probability of positively influencing the others. This work adds to the growing body of literature on what constitutes a bad death and highlights the high prevalence of these indicators in home care. Future work could build from these findings to expand and explore other potential predictors of a bad death, develop risk profiles, and more critically examine caregiver burden.

## Data Availability

The data are not publicly available, but they are directly available to interRAI fellows and their staff and students. Other researchers can access the data from the Canadian Institute for Health Information for researchers who meet the criteria for access to confidential data. These data represent third party data that are not owned nor collected by the study authors. A data request form can be found here: https://www.cihi.ca/en/access-data-and-reports/make-a-data-request.

## Abbreviations

ADL-SHS: Activities of daily living self-performance hierarchy
CaRE: Caregiver risk evaluation
CI: Confidence interval
COPD: Chronic obstructive pulmonary disease
CPS: Cognitive performance scale
DRS: Depression rating scale
IADL: Instrumental activities of daily living
OR: Odds ratio
QIs: Quality indicators
RAI-HC: Resident assessment instrument for home care
STROBE: STrengthening the reporting of observational studies in epidemiology
